# Hypoxia-inducible factor-2a is associated with ABCG2 expression, histology-grade and Ki67 expression in breast invasive ductal carcinoma

**DOI:** 10.1186/1746-1596-7-32

**Published:** 2012-03-27

**Authors:** Lei Xiang, Zhi-Heng Liu, Qin Huan, Peng Su, Guang-Jun Du, Yan Wang, Peng Gao, Geng-Yin Zhou

**Affiliations:** 1 Department of Pathology, School of Medicine, Shandong University, Jinan, Shandong 250012, People's Republic of China; 2 Department of Hepatobiliary Surgery, Liaocheng People's Hospital and Liaocheng Clinical School of Taishan Medical University, Liaocheng, Shandong, 252000, People's Republic of China; 3 Department of Internal Medicine, Shandong Jiaotong Hospital, Jinan, Shandong 250031, People's Republic of China

**Keywords:** HIF-2a, ABCG2(BCRP), Histology-grade, Ki67, Breast invasive ductal cancer, IHC, Tissue microarray, MDR

## Abstract

**Background:**

Breast cancer is the most common cancer and the leading cause of cancer mortality in women worldwide. Hypoxia is an important factor involved in the progression of solid tumors and has been associated with various indicators of tumor metabolism, angiogenesis and metastasis. But little is known about the contribution of Hypoxia-Inducible Factor-2a (HIF-2a) to the drug resistance and the clinicopathological characteristics in breast cancer.

**Methods:**

Immunohistochemistry was employed on the tissue microarray paraffin sections of surgically removed samples from 196 invasive breast cancer patients with clinicopathological data. The correlations between the expression of HIF-2a and ABCG2 as well as other patients' clinicopathological data were investigated.

**Results:**

The results showed that HIF-2a was expressed in different intensities and distributions in the tumor cells of the breast invasive ductal carcinoma. A positive staining for HIF-2a was defined as a brown staining observed mainly in the nucleus. A statistically significant correlation was demonstrated between HIF-2a expression and ABCG2 expression (p = 0.001), histology-grade (p = 0.029), and Ki67 (p = 0. 043) respectively.

**Conclusion:**

HIF-2a was correlated with ABCG2 expression, histology-grade and Ki67 expression in breast invasive ductal carcinoma. HIF-2a could regulate ABCG2 in breast cancer cells, and could be a novel potential bio-marker to predict chemotherapy effectiveness. The hypoxia/HIF-2a/ABCG2 pathway could be a new mechanism of breast cancer multidrug-resistance.

**Virtual slides:**

http://www.diagnosticpathology.diagnomx.eu/vs/2965948166714795

## Background

Breast cancer is the most commonly diagnosed cancer and the leading cause of cancer mortality in women worldwide. Clinically, breast cancer is a remarkably heterogeneous disease in terms of gene expression, morphology, clinical course, and response to treatment. Traditionally, pathologic determinations of tumor size, lymph node status, endocrine receptor status, and human epidermal growth factor receptor 2 (HER2) status have driven prognostic predictions and, ultimately, adjuvant therapy recommendations for patients with early stage breast cancer. In recent years, many potential new prognostic markers of a biochemical nature have been described for breast cancer. These include steroid-hormone receptors, growth factor receptors, activated proto-oncogenes, and proteolytic enzymes et al.

Hypoxia is an important factor involved in the progression of solid tumors and has been associated with various indicators of tumor metabolism, angiogenesis and metastasis [1]. The presence of widespread hypoxia within tumors has been associated with reduced survival after radiotherapy or chemotherapy. Hypoxia has also been linked to poor outcome in a number of tumors regardless of the treatment modalities used. Karolina Helczynska et al. [2] found a significant association between Hypoxia-Inducible Factor-2a (HIF-2a) protein and adverse prognosis of breast cancer, and no such association was found for Hypoxia-Inducible Factor-1a. In other words, HIF-2a is a possible potential independent prognostic bio-marker of breast cancer.

ABCG2 (ATP-binding cassette sub-family G member 2), or breast cancer resistance protein (BCRP), is an vital trans-membrane transporter which plays an important role in the multidrug resistance (MDR) of breast cancer.

Recently, our research team found that ABCG2 is associated with HER-2 expression, lymph node metastasis and clinical stage in breast invasive ductal carcinoma using immunohistochemistry (IHC) stain on the tissue microarray paraffin sections of surgically removed samples from 196 breast cancer patients with clinicopathological data, which means ABCG2 may be a novel potential prognostic bio-marker which can predict biological behavior, clinical progression, prognosis and chemotherapy effectiveness [3].

Two functional elements in the ABCG2 promoter, the estrogen [4] and hypoxia [5] response elements (HRE), and a peroxisome proliferator- activated receptor g (PPARg) response element upstream of the ABCG2 gene [6] have been shown to control ABCG2 expression. So, it is interesting to explore the expression of these two potential prognostic bio-markers, HIF-2a and ABCG2, and their possible correlations in primary breast cancer.

In the present study, the expression of HIF-2a and ABCG2 was detected by immunohistochemistry using tissue microarray according to immunohistochemical phenotypes and the correlationships between HIF-2a and ABCG2 expression/the clinicopathological data were discussed. We demonstrated a possibility of predictive role of HIF-2a in chemotherapy of breast cancer.

## Materials and methods

### Patients and tissue samples

We retrieved tissue samples from patients with breast invasive ductal carcinoma in the Department of Pathology of Qilu Hospital of Shandong University during July 2007 through December 2008. Formalin-fixed and paraffin-embedded tissue specimens from 196 patients with primary breast cancer were included. All archival hematoxylin and eosin (H&E)-stained slides for each patient were reviewed by two pathologists. For the usage of the clinical materials for research purposes, prior patient content and approval from the Institutional Research Ethics Committee were obtained. All the diagnoses were made following the Pathology and Genetics of Tumors of Breast of the World Health Organization Classification of Tumors [7]. Clinicopathologic classification and staging were determined according to the American Joint Committee on Cancer criteria [8].

The histological grade was assessed using the Nottingham grading system [9], and nuclear grade was evaluated according to the modified Black's nuclear grade [10]. Histological parameters such as histological subtype, nuclear grade and histological grade were evaluated according to H&E-stained slides. Clinical parameters included patients' age, tumor size, lymph node status, clinical stage and biological markers (ER, PR, HER2 and ki67 et al.).

### Tissue microarray

For each H&E-stained slide, two representative areas were selected and the corresponding spots were marked on the surface of the paraffin block. Using a tissue microarray punching instrument, the selected areas were punched out and were placed into the recipient block side by side. Each tissue core was 2 mm in diameter and was assigned with a unique tissue microarray location number that was linked to a database containing other clinicopathologic data.

### Immunohistochemistry (IHC)

The streptavidin-peroxidase-biotin (SP) immunohistochemical method was utilized to study the expression of HIF-2a and ABCG2 in 196 paraffin-embedded breast tissues.

In brief, paraffin-embedded specimens were cut into 4 μm sections and baked at 60°C for 60 min. The sections were deparaffinized with xylenes and rehydrated. Then sections were submerged into EDTA antigenic retrieval buffer in a pressure cooker for 10 min and then cooled at room temperature for 20 min. The sections were treated with 3% hydrogen peroxide in methanol to quench the endogenous peroxidase activity, followed by incubation with normal serum to block nonspecific binding. Mouse monoclonal HIF-2 alpha antibody [ep190b] (1:2000; Abcam company, ab8365, USA) and Mouse monoclonal ABCG2 antibody [BXP-21] (1:50; Abcam Company, ab3380, USA) were incubated with the sections overnight at 4°C; the second antibody was from an SP reagent kit (Zhongshan Biotechnology Company, Beijing, China). After washing, the tissue sections were treated with biotinylated anti-mouse secondary antibody, followed by further incubation with streptavidin-horseradish peroxidase complex for 20 mins. Stained with diaminobenzidine (DAB), the sections were counterstained with hematoxylin. For negative controls, the anti-HIF-2a and anti-ABCG2 antibodies were replaced with PBS.

### Evaluation of immunohistochemical staining

The stained slides were reviewed and scored independently by two observers blinded to the patients' information, and the scores were determined by combining the proportion of positively stained tumor cells and the intensity of staining. Tumor cell proportion was scored as follows [11]: 0 (≤ 10% positive tumor cells); 1 (≤ 30% positive tumor cells); 2 (31-50% positive tumor cells); 3 (51-80% positive tumor cells) and 4 (> 80% positive tumor cells). Staining intensity was graded according to the following criteria: 0 (-, no staining); 1 (+, weak staining = light yellow); 2 (++, moderate staining = yellow brown) and 3 (+++, strong staining = brown). Staining index (SI) was calculated as the product of the staining intensity score and the proportion of positive tumor cells. Using this method of assessment, we evaluated HIF-2a and ABCG2 expression in invasive breast cancer cells by determining the SI, with scores of 0,1, 2, 3, 4, 6, 9 or 12. The optimal cutoff value for high and low expression level was identified: an SI score of ≥ 4 was used to define tumors with high expression of HIF-2a and ABCG2, and an SI score of ≤ 3 was used to indicate low expression of HIF-2a and ABCG2, and the SI score of 0 was used to imply negative expression, respectively.

### Statistical analysis

The chi-square test or Fisher's exact test were used to evaluate the correlation between HIF-2a expression and ABCG2 expression, as well as the clinicopathologic characteristics if appropriate. Statistical Analyses were performed using the statistical software package SPSS 13.0 (SPSS, Chicago, IL). Differences were considered statistically significant for p < 0.05.

## Results

**Figure 1:**
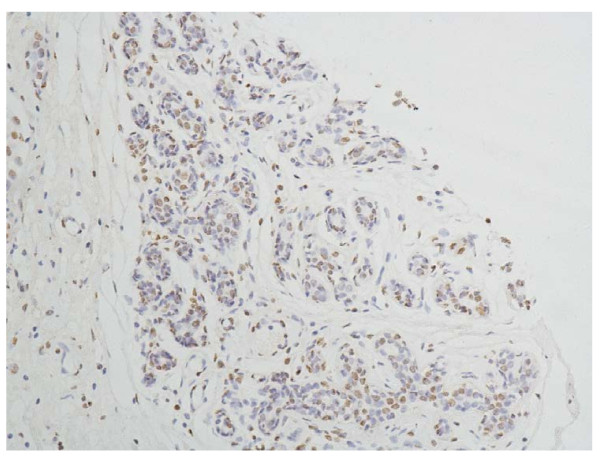
**Normal breast tissue, HIF-2a positive (IHC, SP × 200)**. The HIF-2a staining is weak, and mainly localized mainly in the nucleus of the glandular epithelium cells and vascular endothelium of the breast.

The specificity of the immunodetection was confirmed by using the monoclonal antibodies HIF-2a antibody [ep190b]. A positive stain for HIF-2a was defined as a brown stain observed mainly in the nucleus, and a negative or weak cytoplasmic reactivity observed. Positive staining of normal adjacent ductal epithelia as well as vascular endothelium and stromal cells of the breast has a low level expression (Figure 1). And it can serve as an internal positive control.

**Figure 2:**
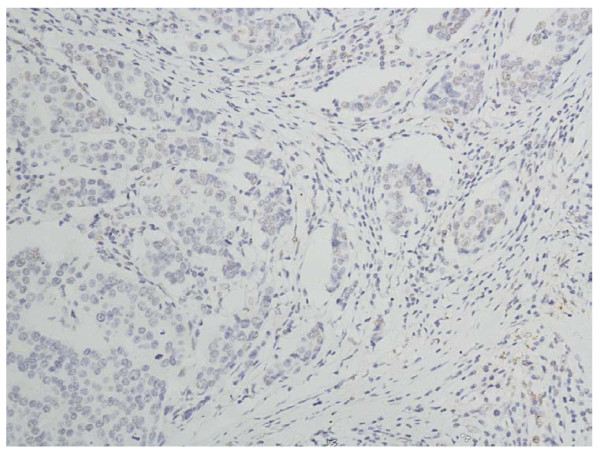
**Breast cancer tissue, HIF-2a negative (IHC, SP × 200)**. The HIF-2a staining is almost negative.

**Figure 3:**
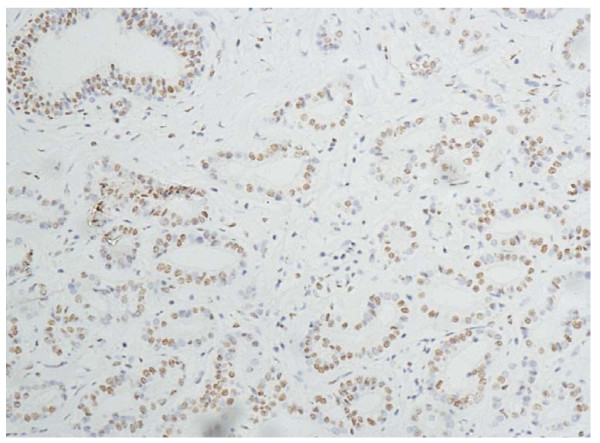
**Breast cancer tissue, HIF-2a positive + (IHC, SP × 200)**. The HIF-2a staining is weak. Left top shows the normal duct of the breast.

**Figure 4:**
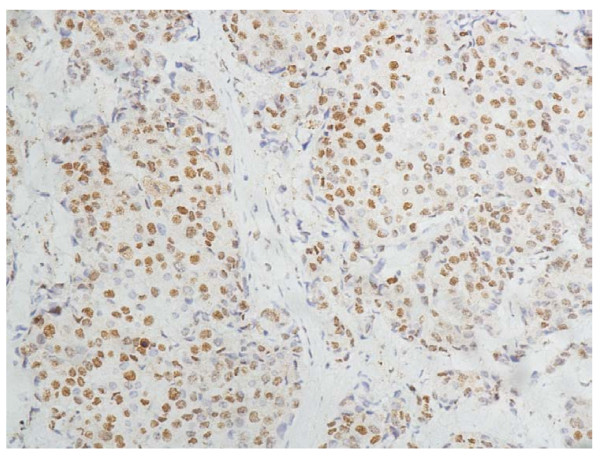
**Breast cancer tissue, HIF-2a positive ++ (IHC, SP × 200)**. The HIF-2a staining is moderate.

**Figure 5:**
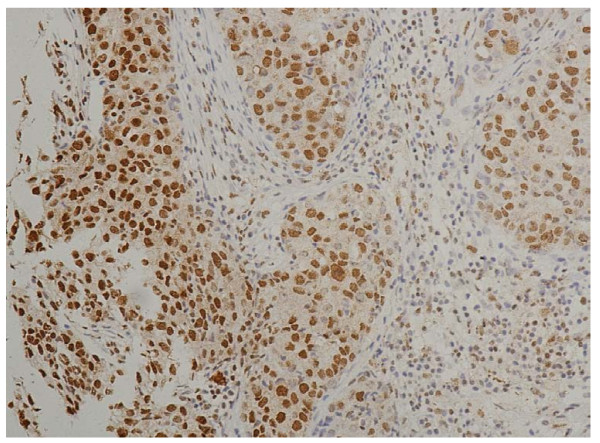
**Breast cancer tissue, HIF-2a positive +++ (IHC, SP × 200)**. The HIF-2a staining is strong.

In the cells of the breast invasive ductal carcinoma, HIF-2a expression was present in different intensities and different cell distributions. Following the staging criteria of stain intensity, 12 cases (6.12%) were identified as completely negative (Figure 2), 78 cases (39.80%) were identified as "+" (Figure 3), 69 cases (35.20%) were identified as "++" (Figure 4), and 37 cases (18.88%) were identified as "+++" (Figure 5). According to the above assessment methods and evaluation criterion, combining the proportion of positively stained tumor cells, the negative, low level and high level expression of HIF-2a was observed in 25 cases (12.76%), 114 cases (58.16%) and 57 cases (29.08%), respectively.

The IHC expression and distribution of ABCG2 in invasive breast cancer cells are as same as the findings of our previous study [3].

There was no significant correlation between the expression level of HIF-2a and biological factors such as patients' age (p = 0.053), histology type (p = 0.285), tumour size (p = 0.601), lymph node metastasis (p = 0.808), ER (p = 0.544), PR (p = 0.343), HER-2 expression (p = 0.923) and clinical stage (p = 0.538). In contrast, statistical analyses indicated that HIF-2a expression was positively related with ABCG2 expression and the correlation was statistically significant (p = 0.001); meanwhile, the correlation between HIF-2a expression and histology-grade/Ki67 was significant (p = 0.029/0.043, respectively). The results are summarized in Table 1.

**Table 1:** The correlation between the expression of HIF-2 alpha and BCRP, the clinicopathological parameter

	Patients n (%)	HIF-2 alpha expression			P value
			
		negative	low	high	
ABCG2 expression					0.001*
negative	26	8	17	1	
low	97	12	58	27	
high	73	5	39	29	

Age					0.053

≤ 50 years	80	10	54	16	

> 50 years	116	15	60	41	

Histology-type					0.285

IDC	155	22	86	47	

IDC with others	41	3	28	10	

Histology-grade					0.029*

G1/G2	143	16	78	49	

G3	53	9	36	8	

Tumour size					0.601

≤2.0 cm	97	13	53	31	

> 2.0 cm	99	12	61	26	

LNM					0.808

-	103	13	58	32	

+	93	12	56	25	

ER					0.544

-/+	96	10	59	27	

++/+++	100	15	55	30	

PR					0.343

-/+	128	14	79	35	

++/+++	68	11	35	22	

HER2					0.923

-/+	138	18	81	39	

++/+++	58	7	33	18	

Ki67					0.043*

Positive ≤50%	154	20	83	51	

Positive > 50%	42	5	31	6	

Clinical stage					0.538

Stage I	60	7	33	20	

Stage IIa and IIb	86	13	47	26	

StageIIIa and IIIb	50	5	34	11	

*P *values were evaluated by chi-square test or the Fisher's exact test. *P < 0.05

## Discussion

HIF-2α, also known as endothelial PAS domain protein 1 (EPAS1) or member of PAS superfamily 2 (MOP2)[12-15], was the second HIF family member to be identified and belonged to the basic helix-loop-helix (bHLH)/Per-ARNT-Sim (PAS) domain family of transcription factors [16]. It activates gene expression via formation of a dimeric complex with HIF-1β (also called aryl hydrocarbon receptor nuclear translocator, ARNT) and subsequent binding to hypoxia response elements (HREs) within target genes. Among its transcription targets are genes involved in proliferation, metabolism, angiogenesis, differentiation, and metastasis [1]. Numerous immunochemical analyses have demonstrated that HIF-2α was over-expressed in a number of primary and metastatic human cancers, and that the level of expression, either as a result of tumor hypoxia or genetic alterations, is correlated with tumor angiogenesis and patient mortality. High HIF-2α expression has been linked to poor patient outcome in several tumor types [17-21].

In the present study, we studied HIF-2α expression in paraffin-embedded tumor samples using the IHC method with the Abcam Mouse monoclonal HIF-2 alpha antibody [ep190b, ab8365], and the positive stain for HIF-2α is located mainly in the nucleus of cells as the product leaflet of the antibody indicates.

In our study, the expression HIF-2α of in most tumor cells (87.24%) of the breast invasive ductal carcinoma is positive, in which about 1/3 cases present relatively high HIF-2α expression in the breast cancer cells. Furthermore, the expression of HIF-2α is correlated with the histology-grade (p = 0.029) and the expression of Ki67 (p = 0.043). Those patients with high expression of HIF-2α were demonstrated to be more frequently showing high histology-grade and high expression of Ki67, which means that HIF-2α have some correlation with worse biological behavior and clinical aggressiveness.

To the best of our knowledge, this is the first report that the expression of HIF-2α is correlated with histology-grade and the expression of Ki67 in the cohort of breast invasive ductual carcinoma. We think it is of great importance for further research. In general, it is consistent with previous literature that high expression of HIF-2α have a worse prognosis than those with low expression. Because there was no case in clinical IV stage in the 196 patients which were used in the study, it is impossible to evaluate the correlation between HIF-2α expression and cases with distant metastasis.

In the study of Helczynska K et al. [2], the expression of HIF-2α is located both in nucleus and cytoplasm of the tumor cells by IHC detection and show a significant correlation to incidence of distant recurrence. We think the different antibodies and clinicopathologic variables used in the two studies may contribute the different results between the study of Helczynska K et al. and ours.

ABCG2 (or BCRP), is the second member of the G subfamily of the ATP-binding cassette (ABC) efflux transporter superfamily that has been the subject of intense study.

In our previous study [3], we found the expression of ABCG2 protein correlated with Her-2 expression, lymph node metastasis and clinical stage in breast invasive ductal carcinoma and ABCG2 could be a novel potential bio-marker which can predict biological behavior, clinical progression, prognosis and chemotherapy effectiveness.

In the present study, we found HIF-2α expression was correlated with ABCG2 expression significantly (p = 0.001) in paraffin-embedded tumor samples using the IHC method. It means that those patients with high expression of HIF-2α were demonstrated to be more frequently showing high immunoreactions with ABCG2, which suggests that HIF-2α may have some correlation with worse chemotherapy effectiveness. The finding is consistent with the study of Cindy M. Martin et al. [22], in whose report HIF-2α was found being a potent transcriptional regulator of the Abcg2 gene, and HIF-2α bond an evolutionary conserved HIF-2α response element (HRE) in the murine Abcg2 promoter. A hypothesis had been raised by Cindy M. Martin et al. that Abcg2 is a direct downstream target of HIF-2α in the side population (SP) progenitor cell population of the adult heart. But in the area of cancer research, there is no such kind of report. To the best of our knowledge, this is the first report that the expression of HIF-2α is correlated with ABCG2 expression in the cohort of breast invasive ductual carcinoma. Our findings could be another evidence for the above hypothesis. And in the area of the mechanism research of breast cancer MDR, our findings pointed out another possible genesis mechanism that hypoxia could induce the expression of ABCG2 by HIF-2α, which promoted the multidrug resistance of breast cancer cells.

Because of the evaluation of IHC staining and samples selecting of tissue microarray, some errors of our study could be possible. Whether HIF-2a is correlated with the prognosis of breast cancer and whether the above possible regulation pathways is correct in cancer cells need to be explored by further study.

## Conclusion

Our study demonstrates the expression of HIF-2α in normal ductal epithelia and in the tumor cells of invasive ductal carcinoma of breast. Its expression is correlated significantly with that of histology-grade and Ki67 in invasive ductal breast cancer. The study also found HIF-2α expression was correlated with ABCG2 expression significantly, which suggests HIF-2α be a potent transcriptional regulator of the Abcg2 gene and Abcg2 be a direct downstream target of HIF-2α in the cells of invasive ductal breast cancer. These findings suggest that HIF-2α is not only one of the hypoxia inducible factors, but also a novel potential bio-marker which could predict worse chemotherapy effectiveness in invasive ductal carcinoma of breast; and indicate that hypoxia promoted the multidrug resistance of breast cancer cells by hypoxia/HIF-2α/ABCG2/MDR pathway.

Further investigation on the molecular mechanism of possible regulation relationships between them should be done.

## Competing interests

The authors declare that they have no competing interests.

## Authors' contributions

LX and YW did the immunohistochemical analysis. PS and PG reviewed all the pathological slides. ZL and QH made the tissue microarray. GD analyzed the data. GZ designed the study. LX drafted the manuscript. All authors read and approved the final manuscript.
